# A Novel Multi-Sensor Environmental Perception Method Using Low-Rank Representation and a Particle Filter for Vehicle Reversing Safety

**DOI:** 10.3390/s16060848

**Published:** 2016-06-09

**Authors:** Zutao Zhang, Yanjun Li, Fubing Wang, Guanjun Meng, Waleed Salman, Layth Saleem, Xiaoliang Zhang, Chunbai Wang, Guangdi Hu, Yugang Liu

**Affiliations:** 1 School of Mechanical Engineering, Southwest Jiaotong University, Chengdu 610031, China; meng_guanjun@my.swjtu.edu.cn (G.M.); wldsalman6@gmail.com (W.S.); Leo_792004@yahoo.com (L.S.); ghu@home.swjtu.edu.cn (G.H.); 2 School of Information Science & Technical, Southwest Jiaotong University, Chengdu 610031, China; liyanjun@my.swjtu.edu.cn (Y.L.); fbwwang@163.com (F.W.); zxl627905@163.com (X.Z.); 3 Department of Industrial & Manufacturing Systems Engineering, Iowa State University, Ames, IA 50011, USA; chbwang@iastate.edu; 4 School of Transportation & Logistics, Southwest Jiaotong University, Chengdu 610031, China; liuyugang@swjtu.edu.cn

**Keywords:** multi-sensors, information fusion, adaptive Kalman filter, particle filter, low-rank representation, vehicle reversing control

## Abstract

Environmental perception and information processing are two key steps of active safety for vehicle reversing. Single-sensor environmental perception cannot meet the need for vehicle reversing safety due to its low reliability. In this paper, we present a novel multi-sensor environmental perception method using low-rank representation and a particle filter for vehicle reversing safety. The proposed system consists of four main steps, namely multi-sensor environmental perception, information fusion, target recognition and tracking using low-rank representation and a particle filter, and vehicle reversing speed control modules. First of all, the multi-sensor environmental perception module, based on a binocular-camera system and ultrasonic range finders, obtains the distance data for obstacles behind the vehicle when the vehicle is reversing. Secondly, the information fusion algorithm using an adaptive Kalman filter is used to process the data obtained with the multi-sensor environmental perception module, which greatly improves the robustness of the sensors. Then the framework of a particle filter and low-rank representation is used to track the main obstacles. The low-rank representation is used to optimize an objective particle template that has the smallest L-1 norm. Finally, the electronic throttle opening and automatic braking is under control of the proposed vehicle reversing control strategy prior to any potential collisions, making the reversing control safer and more reliable. The final system simulation and practical testing results demonstrate the validity of the proposed multi-sensor environmental perception method using low-rank representation and a particle filter for vehicle reversing safety.

## 1. Introduction

Vehicles have long been widely utilized around the world. To reduce the probability and rate of traffic accidents is always the focus of research [1,2,3,4]. The growing number of reversing traffic accidents has become a serious social safety problem in recent years. Collision, especially reversing collision due to careless or violent driving, is one of the major causes of traffic accidents [5,6]. Drivers’ abnormal behavior and some minor mistakes may result in a great deal of accidents when the vehicle reverses. In 2012, the National Highway Traffic Safety Administration (NHTSA) reported that about 8% of traffic accidents were caused by driving in reverse [7]. In Finland, analysis of data indicated that reversing accidents contributed to approximately 24% of motor vehicle crashes and most of the reversing accidents (70%) took place in parking lots [8]. Statistics also show that reversing accidents are 1.2 times more likely to occur with middle-aged drivers than with young drivers. The main reason for the increase of reversing accidents might be that the rear view is a blind and restricted area [8,9]. Also, in another study of the NHTSA [10], 99% of reversing accidents were due to drivers’ behavior and about 80% of all driving mishaps are due to drivers’ inattention when reversing. The reversing accidents reflect problems in vehicle maneuvering and drivers’ observations. In reversing traffic safety, an obstacle detection and avoidance system plays an important role in preventing reversing collisions. The earlier an obstacle in the rear area is detected or the better the restricted rear area is viewed, the more chances there are to protect passengers.

To avoid these reversing accidents and to reduce losses through reversing accidents, several emerging technologies were rapidly developed [11]. Sensors installed at the rear of a vehicle can effectively help watch for potential reverse collisions. Vehicle reversing safety using multi-sensors and pre-collision technology have been extensively developed and implemented. We mainly devote this paper to multi-sensors and key information processing in efforts to enhance safety while reversing.

### 1.1. Multi-Sensor Environmental Perception for Vehicle Reversing

Pre-collision detection using multi-sensor technology, both moving object detection [1] and the vehicle parking assistant [9,12,13,14], is an important part of a reversing vehicle’s active safety system. The systems use multiple sensors, such as RADAR, LIDAR, or GPS technology and a camera, to perceive the current traffic situation for vehicle collision detection and reversing safety [11]. Another example of multiple sensors, including motion sensors (accelerometer, gyroscope, and magnetometer), wireless signal strength indicators (WiFi, Bluetooth, and Zigbee), and visual sensors (LIDAR and camera), is used in a hidden Markov model (HMM) framework for mobile-device user-positioning [15]. A new integrated vehicle health maintenance system (IVHMS) [16], equipped with gear sensors, engine sensors, and fuel and electrical sensors, is reported. The different kinds of sensors mentioned in the above three papers perceive different information and so provide the whole state of the system. Some information, for example-images of a target and the distance between two objects, can be obtained by different sensors. As described in [17], the authors presented multi-sensors of eight thermal infrared and panchromatic images to gain better results than an individual sensor. All of the endeavors of the researchers aim to improve the performance of environmental perception. The authors of [18] described a probabilistic analysis of dynamic scenes and collision risk assessment to improve driving safety by means of sensor data (LIDAR-based sensors). Among the multiple sensors, stereo vision stands out because it is not limited to plane vision and gives precise measurements. Stereo vision can combine the views from between the left and right cameras to obtain a 3D visual of an object [19]. Owing to these features, stereo vision can be used to detect object distance and track obstacles and potential collisions for a reversing or parking vehicle [11,13,20,21]. 

According to [15], multiple sensors might offer erroneous or inconsistent information. So, in order to gain the ability to identify and eliminate spurious data with high accuracy, information from multiple sensors must be processed properly [22]. However, since the different sensors are used to measure different physical phenomena, it is not easy to effectively fuse the information to have a better result. One of the major problems in multi-sensor information fusion is that the sensors frequently provide spurious observations that are useless to predicting and modeling. Thus many researchers have attempted to settle this problem by developing fusion systems and a fusion framework [23] based on the information obtained from multi-sensors. In [24], the authors presented a unified sensor fusion strategy based on a modified Bayesian approach that can automatically identify the inconsistency in sensor measurements. Other authors presented a sensor-fusion module with integrated vision, Global Positioning Systems (GPSs), and Geographical Information Systems (GIS) [23]. GPS and GIS provide prior knowledge about the road for the vision module. Except for positioning systems, information fusion can be used in different fields. A human detection system that can be employed on board for autonomous surveillance was proposed in [25] based on the fusion of two sensor modules, one for a laser and another for visual data. A consensus-like distributed fusion scheme [26] is presented for multiple stationary ground targets by a group of unmanned aerial vehicles with limited sensing and communication capabilities. Some more popular methods for multi-sensor fusion are explored extensively in the literature, including fuzzy logic [27], neural network [28], genetic algorithm [29], and Bayesian information fusion [30].

### 1.2. Target Recognition and Tracking of Vehicle Safety 

Target recognition and tracking plays an irreplaceable role in vehicle reversing safety. The fundamental problem is how to recognize and track the target in a changing environment while the driver is reversing. 

In the process of target recognition and tracking, overcoming contradictions among the target tracking rapidity, precision, and robustness [31] is always the focus. For real-time target tracking using MAVs, paper [32] concentrates on the development of a vision-based navigation system. It is proven to be a realistic and cost-effective solution. On the other hand, some authors presented a robust feature matching-based solution to real-time target recognition and tracking [33] under large-scale variation using affordable memory consumption. The results show that the method preforms well. Other researchers were devoted to designing an experimental setup to have human-robot interaction [34] in a surveillance robot. This is helpful in dangerous or emergent situations, such as earthquake and fire, for tracking the targeted person in a robust manner indoors and outdoors under different light and dynamic conditions.

Moving object detection by a backup camera mounted on a vehicle was proposed in [1]. The authors presented a procedure using traditional moving object detection methods for relaxing the stationary camera’s restriction, by introducing additional steps before and after the detection. The target application was to use a road vehicle’s rear-view camera systems. Unlike the algorithm in paper [1], the authors in [35] used the frame difference method to recognize a regular moving target and the Camshift algorithm to track a significant moving target. In fact, in the application of target recognition and tracking of a vehicle, there is more than one target that needs to be recognized and tracked. This increases the work burden of the system. In [36], a multi-target tracking algorithm aided by high-resolution range profile (HRRP) was proposed. The problem of multi-target data association was simplified to multiple sub-problems of data association for a single target. Similarly, a preceding vehicle detection and tracking adaptive to illumination variation in night traffic scenes was presented based on relevance analysis in the literature [4]. The test results indicate that the proposed system could detect vehicles quickly, correctly, and robustly in actual traffic conditions with illumination variation, which was helpful for vehicle safety. A performance evaluation of vehicle safety strategies for reversing speed was proposed [5]. In [9], a vision-based top-view transformation model for a vehicle parking assistant was presented. A novel searching algorithm estimates the parameters that are used to transform the coordinates from the source image. Using that approach, it is not necessary to provide any interior and exterior orientation parameters of the camera for a parking assistant. Visual sensor-based road detection for field robot navigation was proposed in [18]. The authors presented a hierarchical visual sensor-based method for robust road detection in challenging road scenes. The experimental results show that the proposed method exhibits high robustness. The vision system of a surface moving platform is an important piece of equipment for avoidance, target tracking, and recognition. Paper [37] mainly discussed the feature extraction and recognition methods of multiple targets. In this paper, a goal down image detection adaptive sparse representation and tracking method based on image is proposed. The results show that recognition can achieve over than 90% which means it has a good performance. In [38], the authors presented a stereo vision-based vehicle detection system on the road using a disparity histogram. Their system can be viewed as three main parts: obstacle detection, obstacle segmentation, and vehicle detection, with a 95.5% average detection rate. 

Based on sparse expression, researchers built a new adaptive sparse expression [39] theory system, improved the robustness of the image recognition, and promoted target tracking technology innovation. This system has wide application potential. Some robust visual tracking and vehicle classifications have been proposed in [40,41,42,43]. 

### 1.3. Reversing Speed Control for Vehicle Safety

In [44], model predictive control (MPC) was used to compute the spacing-control laws for transitional maneuvers of vehicles. Drivers may adapt to the automatic braking control feature available on adaptive cruise control (ACC) in ways unintended by designers [45]. In [46], an autonomous reverse parking system was presented based on robust path generation and improved sliding mode control for vehicle reversing safety. Their system consists of four key parts: a novel path-planning module; a modified sliding mode controller on the steering wheel; image processing and real-time estimation of the vehicle’s position; and a robust overall control scheme. The authors of [14] presented a novel vehicle speed control method based on driver vigilance detection using EEG and sparse representation. The scheme mentioned in this paper has been implemented and successfully used to reverse the vehicle. In [47], a Bayesian network is used to detect human action to reduce reversing traffic accidents. The authors used Lidar and wheel speed sensors to detect environmental situations. In [48], a robust trajectory tracking for a reversing tractor trailer system was proposed. They treat the vehicle reversing speed control by virtue of neural network [49], fuzzy control [50], and human–automation interaction [18,51].

Despite successful utilization of the existing approaches and systems, a variety of factors in vehicle reversing safety systems still challenge researchers. Many studies have been conducted on reversing safety systems, focusing on three main problems: (1) how to find smaller size, higher reliability, and lower cost multi-sensors that are suitable to environmental perception; (2) how to realize target recognition and tracking based on information fusion for different physical phenomena measured by the multi-sensors; (3) how to realize the vehicle reversing speed control strategy based on multi-sensor environmental perception and object tracking for preventing collisions in realistic conditions? 

In this paper, we introduce a multi-sensor environmental perception method using low-rank representation and a particle filter for vehicle reversing safety. A multi-sensor environmental perception module based on a binocular-camera system and ultrasonic range finders is used to acquire the distance of an obstacle behind the vehicle. The information fusion algorithm using adaptive Kalman filter is employed to process the data obtained by the binocular vision and ultrasonic sensors. The framework of particle filter and low-rank representation is used to track the main obstacles. After obstacle detection and tracking, the vehicle reversing control strategy takes steps to avoid reversing collisions.

The rest of the paper is organized as follows. In Section 2, we present the general system architecture of our proposed system. Section 3 focuses on multi-sensor environmental perception for vehicle reversing. Target recognition and tracking are developed in Section 4, and vehicle reversing speed control strategies are described in Section 5. Section 6 is devoted to the system simulation and validation. Finally, some conclusions are provided in Section 7.

## 2. System Architecture

**Figure 1:**
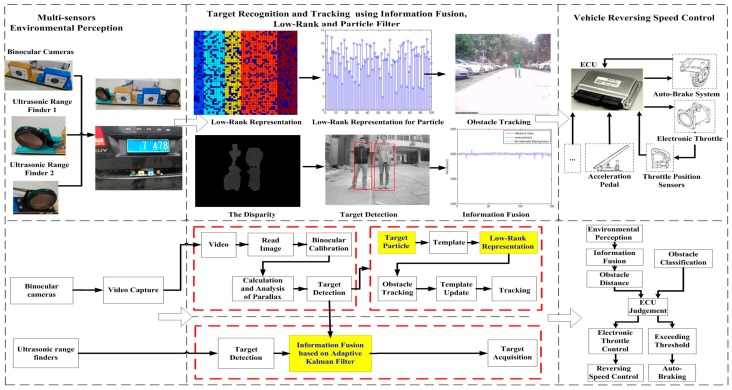
Flowchart of the proposed system.

The general architecture of our system, as shown in Figure 1, is made up of multi-sensor environmental perception, target recognition and object tracking, and vehicle reversing speed control strategy.

In the first step, when a vehicle is reversing, the Electronic Control Unit (ECU) receives the reversing information behind the vehicle and automatically operates the multi-sensors’ environmental perception module to acquire rear-view information. Then the binocular cameras and two ultrasonic range finders capture information about the complex reversing environment. After obtaining the images, we can get the obstacle’s distance information using disparity computation and triangulation. At the same time, two ultrasonic sensors are applied for rear collision intervention, which inform drivers of distance feedback for obstacles behind the vehicle. The left block of Figure 1 shows the binocular cameras and two ultrasonic sensors.

**Figure 2:**
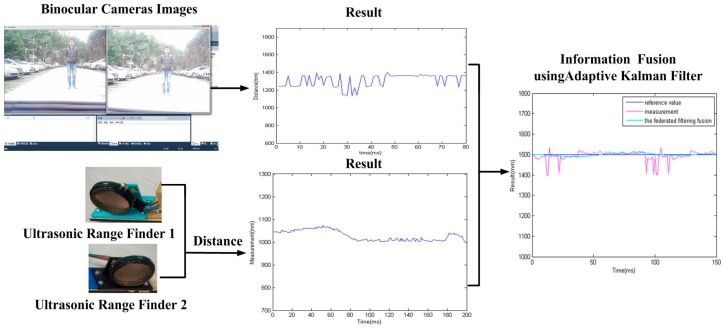
Process of information fusion.

**Figure 3:**
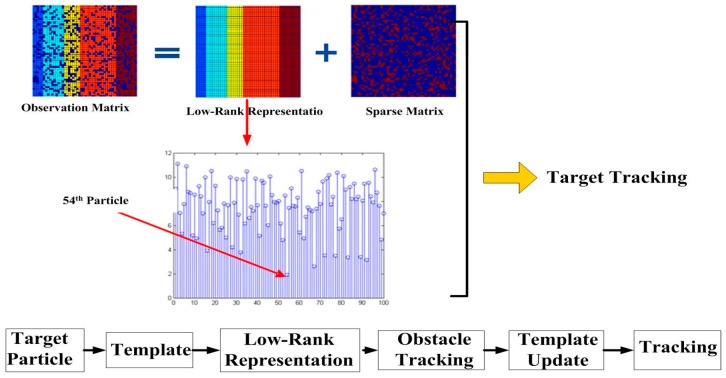
A novel target tracking algorithm in our proposed approach.

The second step includes the information fusion algorithm based on multi-sensors for obstacles detection, and target tracking using particle filter and low-rank representation. As shown in the middle blocks of Figure 1 and Figure 2, an adaptive Kalman filter is used to process the data by binocular vision and ultrasonic sensors. The result of multi-sensor information fusion is very important for a vehicle to keep a safe distance from obstacles. A novel framework of a particle filter based on low-rank representation is used to track the main obstacles for vehicle reversing, as shown in the middle block of Figure 1. In this paper, we introduce a low-rank matrix in the particle filter to choose an optimal objective particle template with the smallest L-1 norm. As shown in Figure 3, combining low-rank representation in a target particle, we eventually choose the particle that has the smallest difference compared with target templates in the candidates set. The proposed novel obstacle tracking algorithm can successfully track the obstacle when the vehicle is reversing.

The final step is the vehicle reversing speed control strategy, also shown in the right block of Figure 1. After target recognition and tracking, the ECU of the vehicle controls reversing speed for vehicle safety. Based on the information fusion of multi-sensor environmental perception and obstacle tracking, ECU will judge the safe distance for reversing. If a danger is detected, ECU will control the speed of the vehicle to avoid a reversing collision.

## 3. Multi-Sensor Environmental Perception

**Figure 4:**
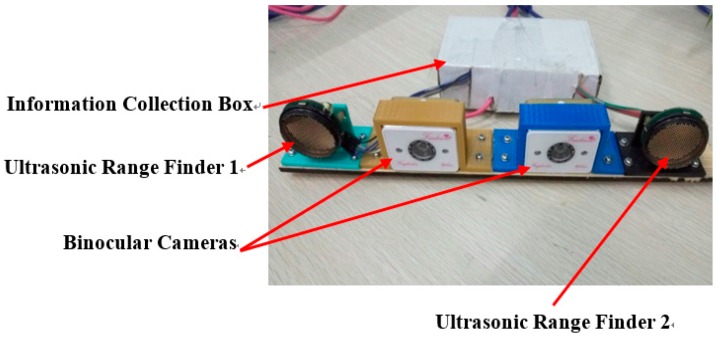
Installation board of multi-sensors.

**Figure 5:**
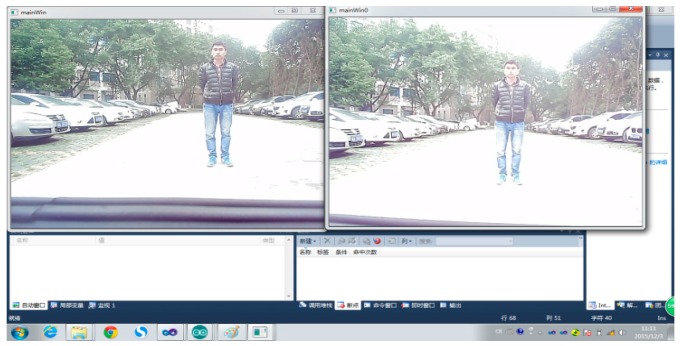
The results of binocular cameras when a vehicle is reversing.

In this section, two types of sensors, ultrasonic range finders and binocular cameras, are used to perceive the environment around a vehicle. As shown in Figure 4, the proposed multiple sensors are used in our research. Two ultrasonic range finders measure the distance from obstacles, and the binocular cameras are used to capture vision and distance information about the obstacles. We integrate the two sensors on a board as a vehicle reversing multi-sensor in Figure 4. The images from the binocular cameras are shown in Figure 5.

### 3.1. Obstacle Detection Based on Binocular Cameras

Stereo vision technology is used more often in machine vision. With two images from left and right, a person can approximately obtain an object’s 3D geometry, distance, and position. In this paper, videos captured by binocular cameras as in Figure 4 are used not only for visual information for a driver, but also for ECU for vehicle reversing safety. 

#### 3.1.1. Binocular Stereo Calibration

In order to get the depth of an obstacle, key parameters are introduced to solve the 3D depth computation of a target for binocular stereo rectification [52] of the binocular cameras. To get these parameters, images are calibrated using methods given in the literature [5]. For binocular stereo rectification, the parameters are defined as follows:

**M**: intrinsic matrix, a 3 × 3 matrix containing camera normalized focal length and optical center.
$f_{x}$, $f_{y}$: camera normalized focal length.
$c_{x}$, $c_{y}$: camera normalized optical center.
**d**: distortion vector, it is a 5 × 1 vector.
$k_{1}$, $k_{2}$, $k_{3}$: radial distortion parameters.
$p_{1}$, $p_{2}$: tangential distortion parameters.
**R**: rotation matrix, it is a 3 × 3 matrix that contains three 3 × 1 vectors.
$r_{1}$, $r_{2}$, $r_{3}$: rotation matrix vectors.
**t**: translation vector, it is a 3 × 1 vector of three translation parameters.
$T_{x}$, $T_{y}$, $T_{z}$: translation parameters.

We can find the relation of the real-world plane coordinates (*X*, *Y*) and the camera coordinates (*x*, *y*) using the above parameters:

(1) $$\left[\begin{array}{l} x\\ y\\ 1 \end{array}\right]=s\left[\begin{array}{ccc} f_{x} & 0 & c_{x}\\ 0 & f_{y} & c_{y}\\ 0 & 0 & 1 \end{array}\right]\left[\begin{array}{ccc} r_{1} & r_{2} & t \end{array}\right]\left[\begin{array}{c} X\\ Y\\ 1 \end{array}\right]$$

where *s* is a scale ratio and **r_3_** can be removed by the depth *Z* = 0, and the coordinate should be modified by a Taylor series expansion at *r* = 0.

(2) $$\begin{array}{l} \left[\begin{array}{c} x_{corrected}\\ y_{corrected} \end{array}\right]=(1+k_{1}r^{2}+k_{2}r^{4}+k_{3}r^{6})\left[\begin{array}{c} x_{d}\\ y_{d} \end{array}\right]\\ +\left[\begin{array}{c} 2p_{1}x_{d}y_{d}+p_{2}(r^{2}+2x_{d}{}^{2})\\ p_{1}(r^{2}+2y_{d}{}^{2})+2p_{2}x_{d}y_{d} \end{array}\right] \end{array}$$

where ($x_{d}$, $y_{d}$) is a coordinate before correction and ($x_{corrected}$, $y_{corrected}$) is a corrected coordinate for stereo rectification. A single camera can be calibrated using Equations (1) and (2).

At the same time, another two parameters called rotation matrix **R**_s_ = ($r_{s1}$, $r_{s2}$, $r_{s3}$) and translation vector **T**_s_ = ($T_{sx}$, $T_{sy}$, $T_{sz}$), which aligns with two cameras, are computed as in Equation (3):

(3) $$P_{l}=R_{s}{}^{T}(P_{r}-T_{s})$$

where $P_{r}$ and $P_{l}$ are the right and left camera coordinates, respectively. A 3D point *P* can be projected into the left and right cameras as in Equations (4) and (5) according to the above single camera calibration:

(4) $$P_{l}=R_{l}P+T_{l}$$

(5) $$P_{r}=R_{r}P+T_{r}$$

where $R_{l}$ is the rotation matrix of left camera and **R*_r_*** is the rotation matrix of right camera. $T_{l}$ is the translation vector of left camera and $T_{r}$ is the translation vector of right camera. $R_{s}$ and $T_{s}$ can be calculated using Equations (3)–(5).

#### 3.1.2. Binocular Stereo Rectification and Stereo Correspondence

When the binocular camera images are obtained, the next steps are to rectify binocular stereo and establish stereo correspondence. Stereo rectification is used to remove the distortions and turn the stereo binocular images into a standard aligned form utilizing the calibration results. This is an important step for calculating the disparity of binocular images for vehicle reversing. Open Source Computer Vision Library (OpenCV) [53] is introduced to rectify the binocular images.

For binocular stereo rectification and stereo correspondence, the parameters are defined as follows:

$R_{s}$: the segment which is used to obtain the minimization of reprojection distortion
$R_{l}$ and $R_{r}$: the rotation matrices
$M_{l}$ and $M_{r}$: intrinsic matrices.

After finding the correspondence points, the disparity can easily be calculated. Disparity optimization algorithm is also used to remove bad matching points.

After stereo rectification and correspondence, we compute the disparity of the binocular images. The disparities of different targets that have a different distance to the binocular cameras usually contain the edge information of the targets, which can be used for separating targets from others. This important characteristic can be used for target recognition and tracking.

The disparity is usually affected by the noise of light and shelter. We must take measures to decrease the noise to obtain the edge information successfully. In our paper, some operations, such as dilation, erosion, and image binarization, are used to settle this problem. After that, there are still regions with a small area that is the noise, so we choose the largest region as the target to track. In order to gain the location of the target, the external rectangle of the target can be obtained. As a result, we can also get the location of the external rectangle. The four points of the rectangle are the initial location of the particle filter. Of course, the target chosen by binocular stereo vision is the particle template.

#### 3.1.3. Binocular Triangulation

**Figure 6:**
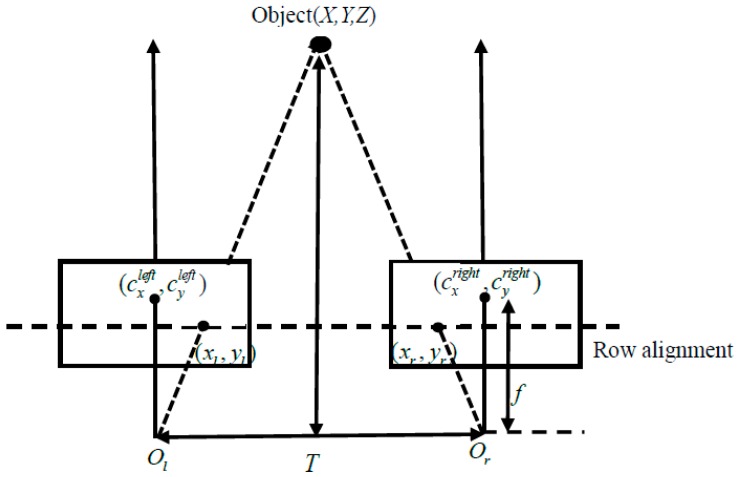
Binocular triangulation model.

After binocular stereo rectification and correspondence, binocular triangulation is used to compute the position of a target in 3D space. A binocular stereo model is shown in Figure 6. The theorems of binocular triangulation are analyzed in Equations (6)–(8):

(6) $$\begin{array}{l} \frac{X-(x_{l}-c_{x}{}^{left})}{X}=\frac{Z-f}{Z}\\ \;\;\Rightarrow X=\frac{Z(x_{l}-c_{x}{}^{left})}{f} \end{array}$$

(7) $$\begin{array}{l} \frac{T-(x_{l}-c_{x}{}^{left})-(c_{x}{}^{right}-x_{r})}{T}=\frac{Z-f}{Z}\\ \Rightarrow Z=\frac{fT}{\left(x_{l}-x_{r})-(c_{x}{}^{left}-c_{x}{}^{right}\right)} \end{array}$$

(8) $$\begin{array}{l} \frac{Y-(y_{l}-c_{y}{}^{left})}{Y}=\frac{Z-f}{Z}\\ \Rightarrow Y=\frac{Z(y_{l}-c_{y}{}^{left})}{f} \end{array}$$

where ($x_{l}$, $y_{l}$), ($x_{r}$, $y_{r}$), ($c_{x}^{left}$, $c_{y}^{left}$), and ($c_{x}^{right}$, $c_{y}^{right}$) are corrected through the above steps. In Equation (7), T is the distance between the binocular cameras’ centers. At the same time, the obstacle’s depth, width, and height can also be obtained by the binocular vision for target detection.

### 3.2. Obstacle Detection Based on Ultrasonic Range Finders

An ultrasonic sensor is used to find the range of a target by means of capturing the reflected ultrasonic wave. An ultrasonic wave is a mechanical vibration at a frequency higher than the sound wave. It is used widely for the high frequency, the short wave length, the lower diffraction, and especially the good direction. However, the range of this sensor is limited to 0–10 m. In our proposed system, two ultrasonic range finders are installed on the rear board for the distance of an obstacle and the instantaneous information. The ultrasonic range finder, KS109, is shown in Figure 4. 

## 4. Target Recognition and Tracking Based on Information Fusion and the Improved Particle Filter

**Figure 7:**
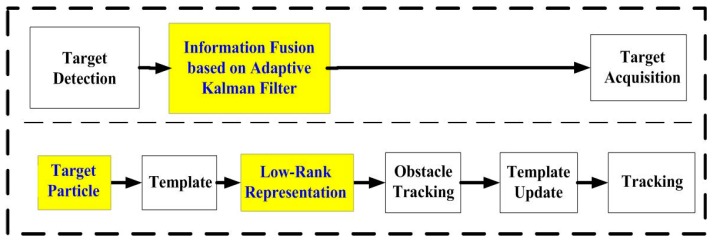
Structure of proposed information fusion and tracking algorithm.

The information fusion algorithm using an adaptive Kalman filter is employed to process the data obtained from binocular vision and ultrasonic sensors of the same obstacle at the same time. It improves the robustness of the sensors. Then the improved particle filter based on low-rank representation tracks the main obstacles. The low-rank representation is used to optimize an objective particle template that has the smallest L-1 norm. This optimization improves the tracking performance. The structure of the proposed algorithm for information fusion and tracking is shown in Figure 7.

### 4.1. Data Fusion Based on Adaptive Kalman Filter

#### 4.1.1. The Basic Principle and Structure of Adaptive Kalman Filter

In the model of a Kalman filter, we use one stochastic differential equation,

(9) X(k)=AX(k−1)+BU(k)+W(k)

where *X*(*k*) represents the state of the system in moment *k* and *U*(*k*) is the control quality of the current state. *A* and *B* are the parameters of the system, which vary in different systems. *W*(*k*) is the processing noise.

Therefore, we can describe the measurement by:

(10) Z(k)=HX(k)+V(k)

where *Z*(*k*) represents the measurement, *H* is the parameter of the measuring system, and *V*(*k*) is the noise in measuring. Based on the model of the Kalman filter, the current state can be estimated based on the previous state. It is described as:

(11) X(k|k−1)=AX(k−1|k−1)+BU(k)

where *X*(*k*|*k* − 1) is the estimation based on the previous state and *X*(*k* − 1|*k* − 1) is the optimal result of the previous state. After updating the system, the covariance can be updated using Equation (12). We use P to represent the covariance:

(12) P(k|k−1)=AP(k−1|}k−1)A′+Q

where covariance *P*(*k*|*k* − 1) corresponds to *X*(*k*|*k* − 1), *P*(*k* − 1|*k* − 1) corresponds to *X*(*k* − 1|*k* − 1), A′ is the transpose of *A*, and *Q* represents the variance of the system. We can have the optimal value *X*(*k*|*k*) using the measurement of the system and the predicted value:

(13) $$\begin{array}{l} X(k|k)=X(k|k-1)\\ \;\;\;\;\;\;\;\;\;\;\;\;+Kg(k)(Z(k)-HX(k|k-1)) \end{array}$$

where *Kg* is the Kalman gain obtained by

(14) Kg(k)=P(k|k−1)H′/(HP(k|k−1)H′+R)

In order to update the system constantly, the covariance updates through

(15) P(k|k−1)=(I−Kg(k)H)P(k|k−1)

where *I* is the identity matrix. Equations (11)–(15) are the basic frame of the Kalman filter.

#### 4.1.2. Information Fusion Based on Federal Kalman Filter

**Figure 8:**
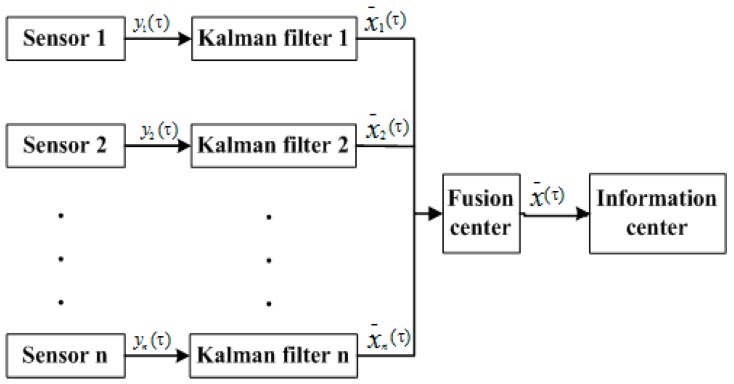
Federal filter information fusion structure.

In this paper, a federated Kalman filter is used for multi-sensor information fusion as in Figure 8. An adaptive federal filter is a decentralized scheme to distribute dynamic information. The dynamic information has two parts, information about the state equation and information about the observation equation.

As shown in Figure 8, the output $x_{i}(\tau )$ and covariance $P_{i}(\tau )$ of the sub-filters in federal filter fusion are local estimations based on the measurement of the subsystem. The outputs, X(τ) and P(τ), determined by all the subsystems of the filter, are the optimal estimation. Their relationship can be described as follows:

(16) $$P(\tau )={\left[\sum_{i}^{N,M}P_{i}^{-1}(\tau )\right]}^{-1}$$

(17) $$\bar{X}(\tau )=\sum_{i=1}^{N,M}P_{i}^{-1}(\tau ){\bar{x}}_{i}(\tau )$$

After fusing, the main filter distributes the information to each sub-filter. The principle of allocation can be described as:

(18) $$P^{-1}(\tau )X(\tau )=\sum_{i=1}^{N,M}P_{i}^{-1}(\tau )x_{i}(\tau )$$

(19) $$P_{i}^{-1}(\tau )=\beta_{i}P^{-1}(\tau )$$

(20) $$Q_{i}^{-1}(\tau )=\beta_{i}Q^{-1}(\tau )$$

(21) $$x_{i}=X(\tau )$$

The parameter $\beta_{i}$ which is shown above, should meet the following requirement:

(22) $$\sum_{i=1}^{N,M}\beta_{i}=1\;\;\;\;\;\;\;\;\;\;i=1,2,\ldots ,N,M$$

Actually, we used four real sensors in our proposed multi-sensor system, including binocular cameras and two ultrasonic rangefinders. The above equations should be changed according to the state equation and measuring equation of the system.

### 4.2. Target Tracking Based on the Modified Particle Filter

#### 4.2.1. Introduction of Particle Filter

***Framework*:** A particle filter based on the Monte Carlo method is widely used in many fields. We can get the state probability using the following steps:

(23) $$p(x_{k}|y_{1:k-1})=\int p(x_{k}|x_{k-1})p(x_{k-1}|y_{1:k-1})dx_{k-1}$$

(24) $$p(x_{k}|y_{1:k})=\frac{p(y_{k}|x_{k})p(x_{k}|y_{1:k-1})}{p(y_{k}|y_{1:k-1})}$$

where $x_{k}$ is a state variable at time *k* and $y_{k}$ is an observation of $x_{k}$, and $p(x_{k}|y_{1:k-1})$ is a normalizing constant in Equation (25):

(25) $$p(y_{k}|y_{1:k-1})=\int p(y_{k}|x_{k})p(x_{k}|y_{1:k-1})dx_{k}$$

By spreading *N* samples at time *k* {*x_ki_*, *i* = 1, ..., *N* }~importance distribution *q(x)* with the weights {*wki*, *i* = 1, ..., *N* }, the weight is approximated in the following:

(26) $$w_{k}^{i}\propto w_{k-1}^{i}\frac{p(y_{k}|x_{k}^{i})p(x_{k}^{i}|x_{k-1}^{i})}{q(x_{k}|x_{1:k-1},y_{1:k})}$$

A maximal approximate optical state is given by the following:

(27) $$x_{k}^{*}=\underset{x_{k}{}^{i}}{\arg\max}\;p(x_{k}{}^{i}|y_{1:k})$$

***Model***: $x_{k}$ contains the affine transformation parameters that convert the obstacle region to a fixed size rectangle. Parameters in $x_{k}$ are independently drawn from a Gaussian distribution around $x_{k-1}$. $y_{k}$ is the transformed image and is stretched into columns of the obstacle region with a constant size by using $x_{k}$. For details about the particle filter, please refer to the literature [5].

#### 4.2.2. Introduction of Low-Rank Representation and Principal Component Analysis

**Figure 9:**
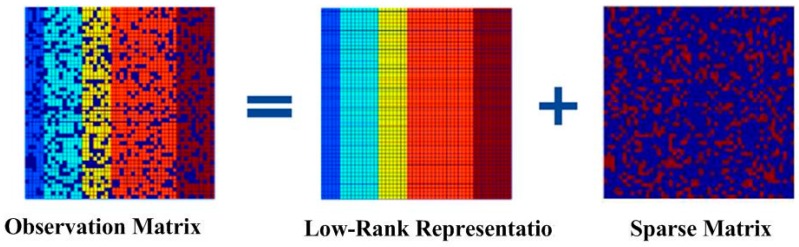
Sparse and low-rank matrix decomposition.

As in many practical applications, the given data matrix D is low rank or approximately low rank. In order to restore the low-rank structure of matrix D, matrix D is decomposed into two unknown matrices, *X* and *E*, as in D = X + E. *X* is low rank, as shown in Figure 9.

(28) D=X+E

(29) $$\min_{X,E}\;\;\;\;rank(X)+\gamma ||E|{|}_{0}\;s.t\;\;\;D=X+E,$$

where $||E|{|}_{0}$ is the number of the nonzero elements. Because this is a non-deterministic polynomial, it needs convex optimization. The formula turns to

(30) $$\min_{X,E}\;\;\;||X|{|}_{*}+\lambda ||E|{|}_{1}\;s.t.D=X+E,$$

where $||X|{|}_{*}$ is the trace nor, and the sum of the singular value of the matrix; $||E|{|}_{1}$ is the L1 norm and the sum of the absolute value of the element, and λ is the weight of the formulation. In this way, we convert the problem of searching for the rank of the matrix into searching for the trace norm of the matrix.

In the process of mathematical optimization, the Lagrange multiplier method is one of the optimization methods that can help with finding the extremum of a multivariate function bounded by one or more variables. The Augmented Lagrange Multiplier (ALM) algorithm also makes use of the multiplier method. The target function is:

(31) $$\begin{array}{l} \underset{D,X,E}{\min}\;\;\;\;l(D,X,E)=\underset{D,X,E}{\min}||X|{|}_{*}+\lambda ||E|{|}_{1}\\ \;\;\;\;\;\;\;\;\;\;\;\;\;\;\;\;\;\;\;\;\;\;\;\;\;\;\;\;\;\;\;\;\;+TR\left\{Y^{T}\left(D-X-E\right)\right\}+\frac{\mu }{2}||D-X-E|{|}_{F}^{2} \end{array},$$

where $||*|{|}_{F}$ is the Frobenius norm. The iterative process of the algorithm is shown in Algorithm 1.

**Algorithm 1** Algorithm of low rank representation**Input****Initialization:**
$E_{0}=Y_{0}=0,k=0$**Procedure** **While**
$||D-X-E|{|}_{F}>10^{-7}||D|{|}_{F}$
**do**   $X_{k+1}=G_{1}/\mu (D-E_{k}-Y_{k}/\mu )$   $E_{k+1}=E_{\lambda }/\mu (D-X_{k+1}-Y_{k}/\mu )$   $Y_{k+1}=Y_{k}+\mu (D-E_{k+1}-X_{k+1})$   k=k+1 **End while****Output X,E**

In the process of iteration, the main computation is a singular value decomposition (SVD). In order to improve the speed of the system, we use IALM in our paper.

#### 4.2.3. Low Rank Representation for Obstacle Recognition and Tracking

As shown in Figure 7, we introduce low-rank representation into the particle filter to improve the tracking performance.

The target template set $D\;=[d_{1},d_{2}\ldots ,d_{n}],\;\;\;D\in R^{m\times n}$ is defined to have *n* target templates and *m* dimensions of the matrix. There are *k* candidate targets generated based on the framework of particle filter. They are defined as:

(32) $$X=\left\{x_{1},x_{2},\ldots ,x_{k}\right\},\;\;X\in R^{m\times k}$$

If we combine each particle of the candidate targets $X\in R^{m\times k}$ with target template $D\in R^{m\times n}$ one by one to form a new matrix $Y_{i}\in R^{m\times \left(n+1\right)}$, we have

(33) $$Y=[D,x_{i}]$$

Then, we can make use of principle component analysis:

(34) $$\min_{Z,E}\;\;\;rank(Z_{i})+\gamma ||E_{i}|{|}_{0}\;s.t.Y_{i}=Z_{i}+E_{i}$$

which is a low-rank matrix obtained after the computation. It can be considered as a matrix that consists of the unchangeable data of the target.

(35) $$Z_{i}=[D\prime ,x_{i}\prime ]$$

where $Y=[D,x_{i}]$ is an observation that can be decomposed into a low-rank matrix $Z_{i}\in R^{m\times \left(n+1\right)}$ and a noise matrix $E_{i}\in R^{m\times \left(n+1\right)}$. For the element of the candidate template set $X=\left\{x_{1},x_{2},\ldots ,x_{k}\right\}$, it will be a zero matrix. Based on the theory described above, Equation (34) can be changed to:

(36) $$\min_{Z,E}\;\;\;||Y_{i}|{|}_{*}+\lambda ||E|{|}_{1}\;s.t.\;\;\;Y_{i}=Z_{i}+E_{i}$$

**Figure 10:**
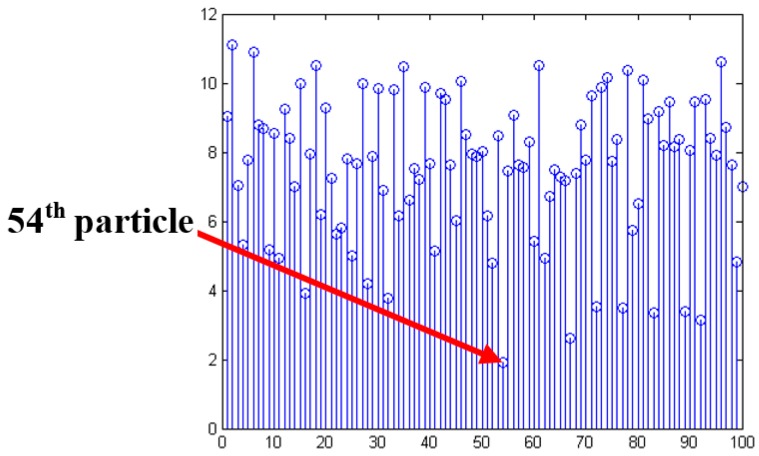
The smallest $||e|{|}_{1}$ of the particles.

In this case, $Z_{i}$ is a low-rank matrix. However, we mainly concentrate on the noise matrix $E_{i}$, because the last column of matrix $E_{i}$ is smaller if the target template set *D* gets closer to the target. We define the last column of $E_{i}$ as $e_{i}$, which represents the difference between the target of the current frame and the sample set. The optimal objective is to have the smallest L-1 norm. Actually, $e_{i}$ can be considered as the difference in the process of tracking of the shelter, and the change of the light. We chose particles having the smallest difference compared with target templates in the candidate set, as shown in Figure 10. The chosen particle has the smallest that is the most desirable particle. It shows that this kind of algorithm can successfully track the target when the vehicle is reversing.

## 5. Vehicle Speed Control Strategy

**Figure 11:**
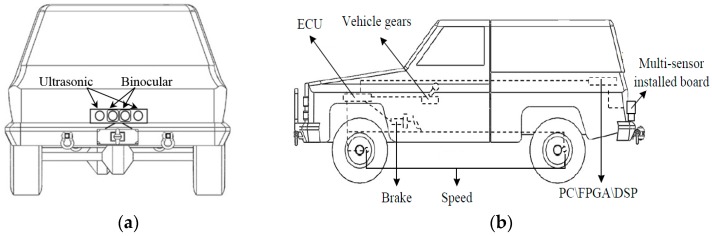
Vehicle reversing speed control prototype: (**a**) position of multi-sensors; (**b**) prototype of the vehicle reversing speed control system.

In this section, we use the vehicle reversing speed control algorithm as in [5] to keep the vehicle in a safety speed. This makes the reversing control safer and more reliable. Table 1 shows strategies of ECU under different conditions for vehicle reversing safety. The fuzzy rules in [5] and Table 1 are presented to show the feasibility. The system is enabled to take control of the electronic throttle opening and automatic braking to avoid collisions. The prototype shown in Figure 11 makes reversing control more reliable [5]. 

## 6. Simulation and Validation

**Figure 12:**
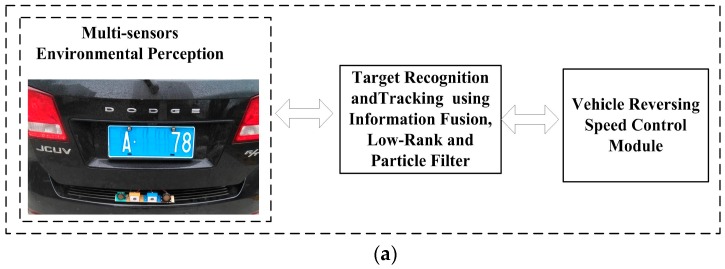

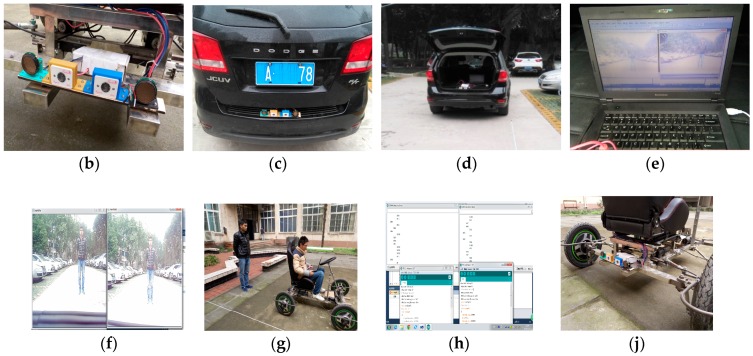
Experimental setup. (**a**) Experimental prototype; (**b**) multi-sensor in proposed system; (**c**) experimental vehicle; (**d**) test preparation; (**e**) test preparation; (**f**) binocular camera images; (**g**) obstacle detection using ultrasonic range finders; (**h**) tests using a homemade vehicle; (**i**) tests using a homemade vehicle.

In this section, experiments have been done to confirm the effectiveness of the system. As shown in Figure 12, a real vehicle reversing experimental environment is used. The ultrasonic sensors work under the control of Arduino chips and the binocular cameras are operated by a laptop under the environment of Microsoft Visual.

There are three parts to our experiment, as shown in Figure 11a. The first part is a multi-sensor that perceives the environment of the vehicle. The vision information is obtained from binocular cameras and the distance information from ultrasonic sensors. The second part is target recognition and tracking using information fusion and low rank with particle filter. Simulation and tests about reversing speed control are the third part.

Figure 12 shows the real experimental environment for testing the system. The algorithm was tested on video sequences captured by the binocular cameras, as shown in Figure 12b, where the binocular cameras were manufactured at our lab. This system has been field-tested on the test vehicle in Figure 12c. Figure 12d–g illustrate the experiments on binocular camera images and obstacle detection using ultrasonic range finders. As shown in Figure 12h,i, in our experiments a homemade experimental vehicle is designed to test our system without traffic risk

The ultrasonic sensors and binocular cameras work simultaneously. Ultrasonic sensors are first used to detect whether there is an obstacle within 0–10 m or not. The binocular cameras capture the visual information at the back of a vehicle at the same time. If an obstacle exists, the system will start to target the obstacle and compute the distance to it based on the binocular stereo vison. After that, the initial location of the target is sent to the particle filter. Finally, the system controls the speed based on target tracking and information fusion.

### 6.1. Experimental Results of Binocular Vision

**Figure 13:**
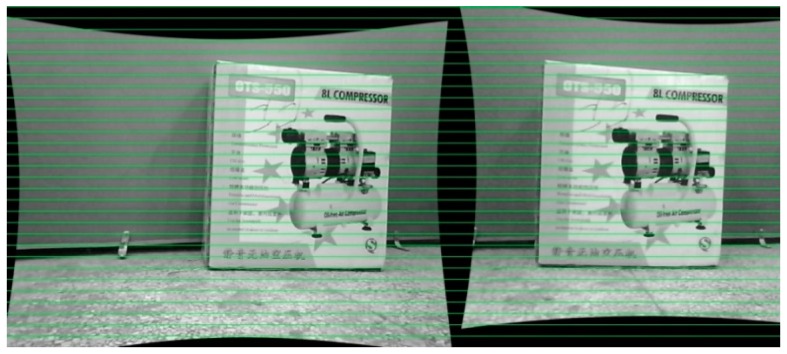
Binocular calibration of our system.

The distance to an obstacle is tested by using the 3D reconstruction of binocular vision, mainly for the depth of the obstacle. Firstly, we use a Matlab box to have the parameters of the binocular cameras, which are then installed into Microsoft Visual for the calibration of images captured from the right and left cameras. The results of calibration are shown in Figure 13. We can see that the images are calibrated properly.

**Figure 14:**
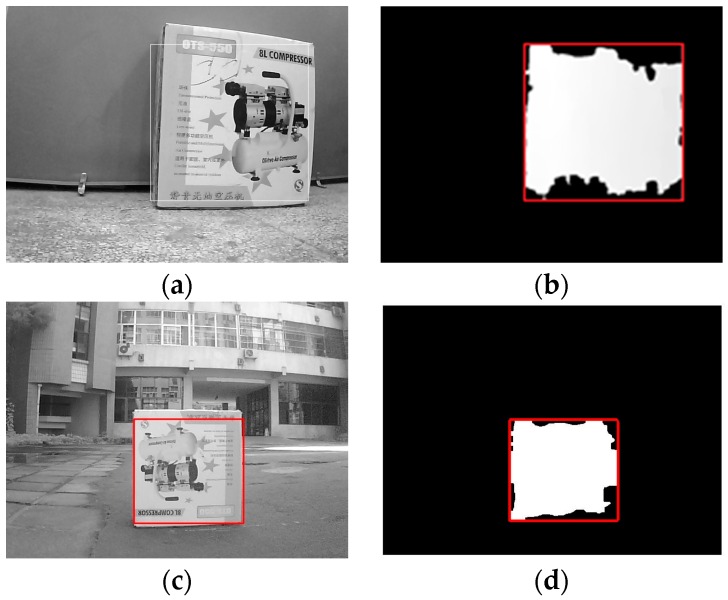
Results of target grab: (**a**) target grabbing from the disparity indoor; (**b**) target detection using images indoor; (**c**) target grabbing from the disparity outdoor; (**d**) target detection using images outdoor.

**Figure 15:**
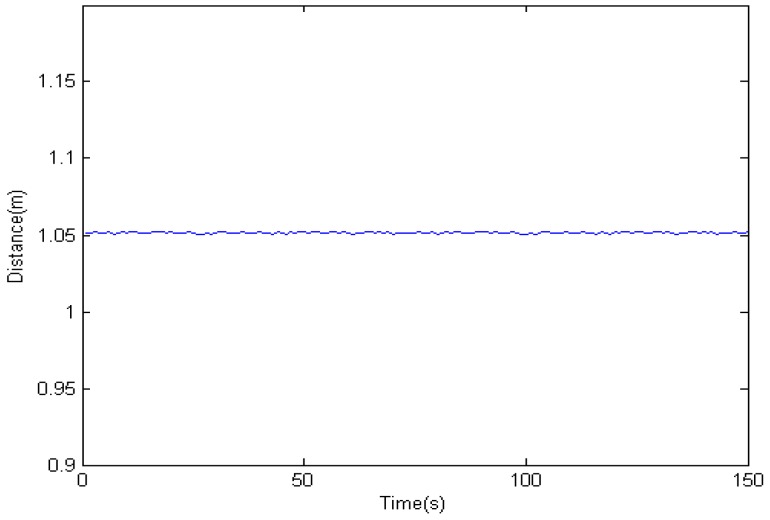
The distance to an obstacle obtained by binocular vision.

**Figure 16:**
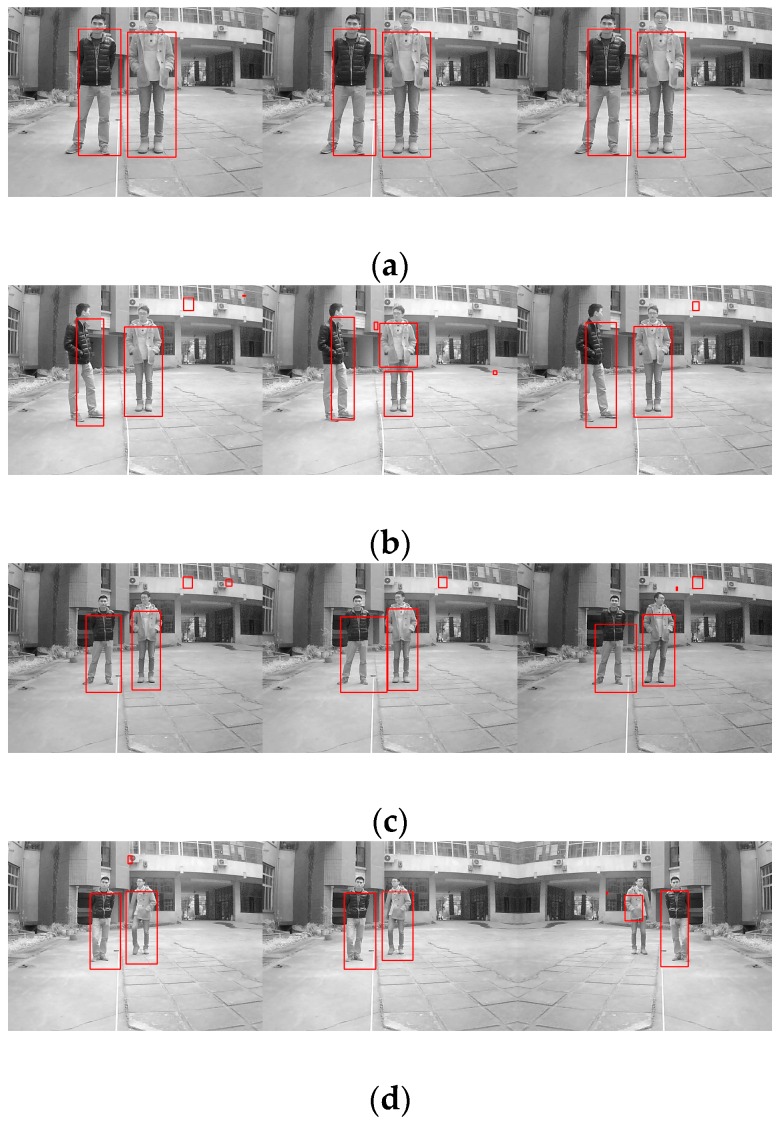
Obstacle detection results: (**a**) Obstacle detection results at 1.0 m; (**b**) Obstacle detection results at 1.0 m; (**c**) Obstacle detection results at 1.0 m; (**d**) Obstacle detection results at 1.0 m.

After the calibration process, stereo rectification and stereo correspondence are made to acquire the disparity of the images. As shown in Figure 14a, the distance to obstacles is obtained from the disparity. In addition, the disparity is used to detect the obstacle. The external rectangle corresponds to the target, as shown in Figure 14b, and the objects are successfully detected. After the disparity of the target, we can calculate the distance to an obstacle based on Equation (6). Here, the distance to an obstacle based on binocular vision is shown in Figure 15. The obstacle detection results at different distances are shown in Figure 16a–d. In Figure 16a, the obstacle distance is about 3.5 m away, and from the detection results it can be seen that the obstacle is well detected. Figure 16b, c show the detection results when the obstacle is about 4.0 m and 5.0 m away, respectively. We can see that the obstacles are well detected. We chose the largest region as the target to track. In Figure 16d, the obstacle is about 6.0 m away, and from the detection results we can see that the obstacle is also well detected. This shows the validity of the proposed binocular vision obstacle detection module.

### 6.2. Information Fusion Based on an Adaptive Kalman Filter

**Figure 17:**
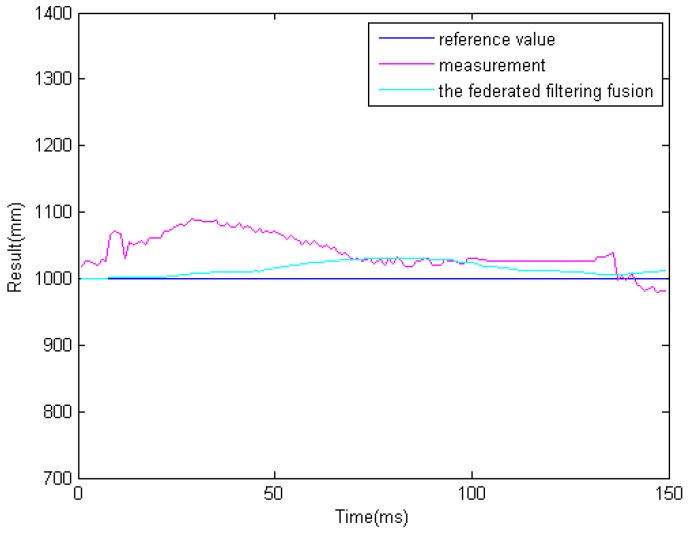
The results of information fusion based on an adaptive Kalman filter using binocular cameras and two ultrasonic sensors at 1.0 m.

**Figure 18:**
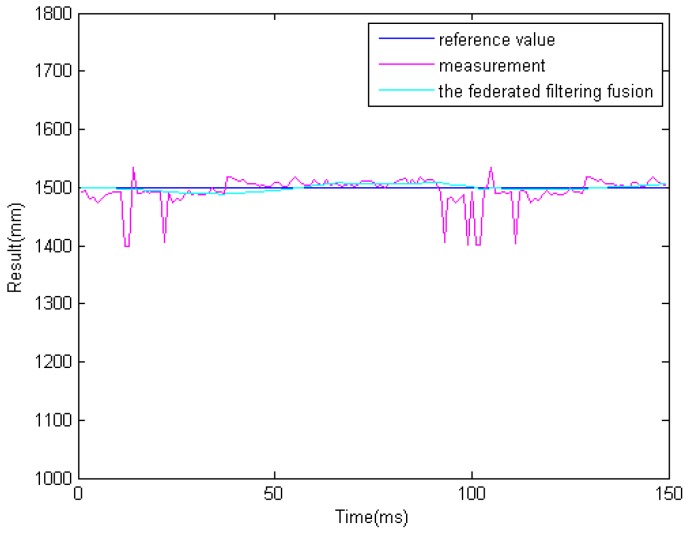
The results of information fusion based on an adaptive Kalman filter using binocular cameras and two ultrasonic sensors at 1.5 m.

**Figure 19:**
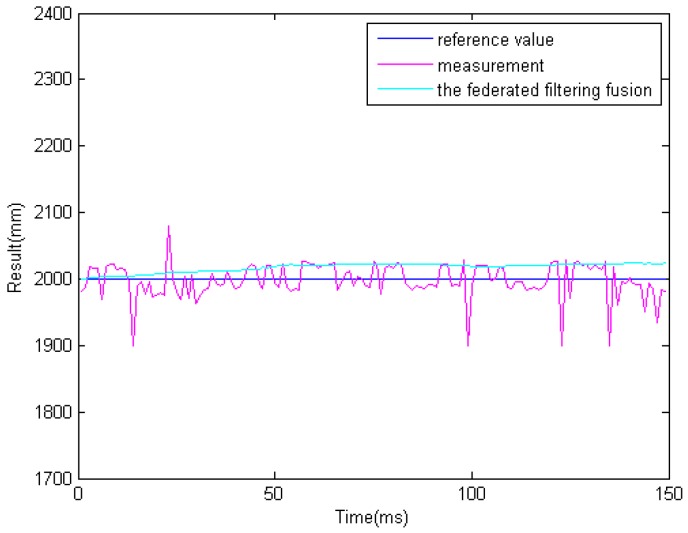
The results of information fusion based on an adaptive Kalman filter using binocular cameras and two ultrasonic sensors at 2.0 m.

In the previous step, we get different results related to the same obstacle from the two ultrasonic sensors and binocular cameras. Because the obstacle data is obtained from four sensors. So it must be processed properly. The results of information fusion based on an adaptive Kalman filter using binocular cameras and two ultrasonic sensors are shown in Figure 17, Figure 18 and Figure 19 at 1.0, 1.5, and 2.0 m. The proposed information fusion algorithm has better performance when the system is influenced by the environment or other factors greatly.

### 6.3. Target Recognition and Tracking Based on the Modified Particle Filter 

**Figure 20:**
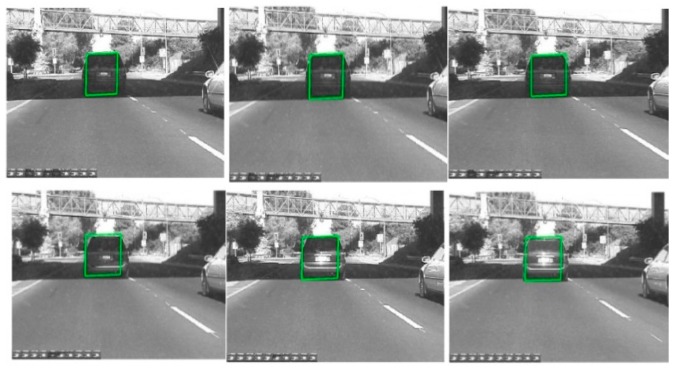
The results of object tracking based on a particle filter using low-rank representation.

**Figure 21:**
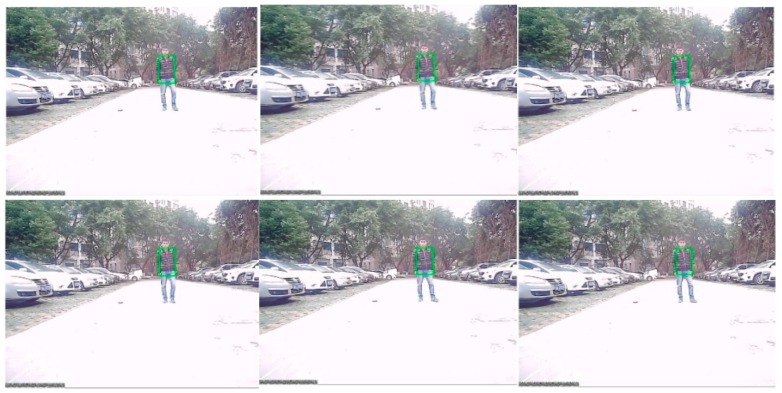
Tracking results based on a particle filter using low-rank representation.

**Figure 22:**
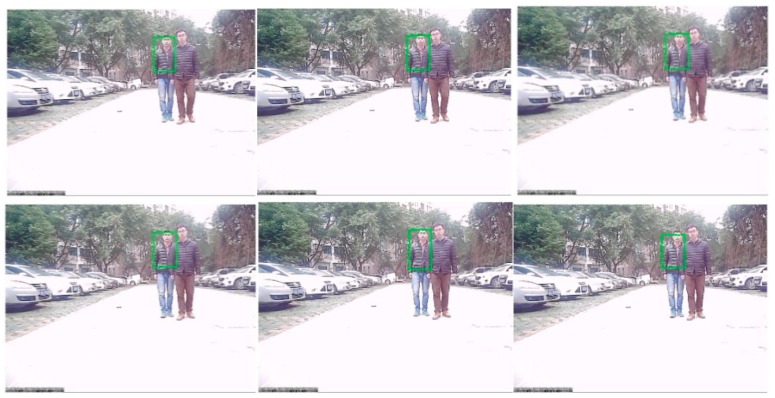
Tracking results based on a particle filter using low-rank representation under a sheltered object.

After the information fusion and target detection, using low-rank representation, the framework of the particle filter tracks and identifies obstacles like human or animal bodies and vehicles, as shown in Figure 20. The proposed algorithm can track any object in various light intensity as well as in the shadows of other objects. In Figure 20, we tried to track and recognize a vehicle, where the target is marked as the green rectangle. It can be seen that even when the vehicle is in the shadows, the target can be well tracked and recognized. Figure 21 shows the tracking results for a human based on a particle filter using low-rank representation, where the human is well targeted. Figure 22 shows a contrast experiment wherein we tried to track and recognize the man on the left in an object’s shadow. From the results, we can see that the selected target is well tracked and recognized successfully.

In order to prove the effectiveness of the system, an experiment about the fps (frames per second) has been done on a computer equipped with an Intel i3-4150 processer at 3.5 GHz and 4 GB memory in the MATLAB 2012a environment. The comparison of fps between our proposed algorithm and the algorithm in the literature [41] is shown in Table 2. As we can see from this table, we use the same video taken by other researchers but with different algorithms. When the number is set to 30, the result of our proposed algorithm can get to 148.1246, about 7 to 8 times that of the algorithm used by other researchers. While we increase the number of frames, it still keeps the same trend. These results indicate that the proposed method shows better performance in obstacle tracking.

On the other hand, we have counted the tracking rate based on the 200 frames of the same video in which the light changes a lot using four videos offered by other researchers and captured by ourselves. Table 3 shows the results of the tracking. As shown in Table 3, the proposed algorithm in our paper can also reach good performance.

### 6.4. Experimental Results of Vehicle Speed Control Based on Multi-Sensor Environmental Perception

**Figure 23:**
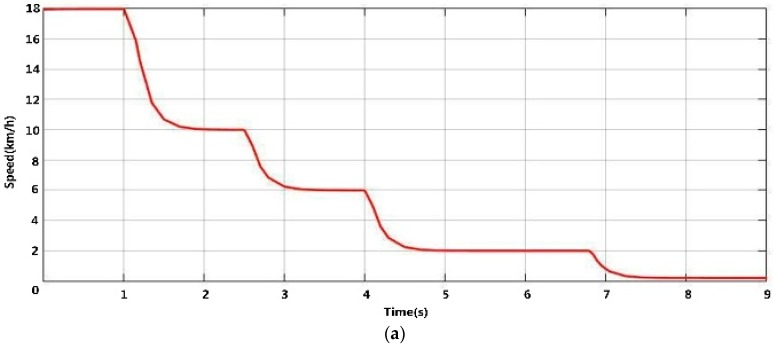

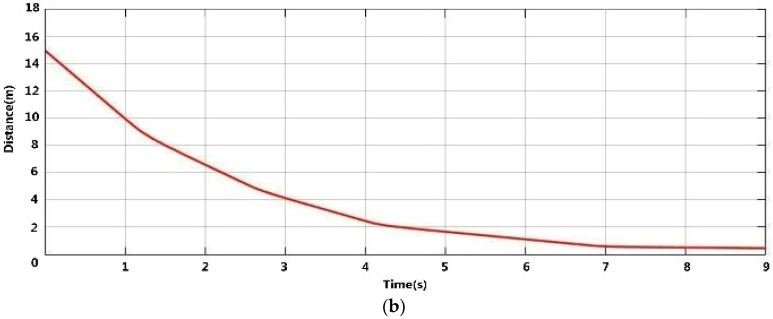
Simulation results of the proposed vehicle reversing speed control. (**a**) Relationship between speed and time; (**b**) Relationship between distance and time.

Figure 23 shows the simulation results of vehicle reversing control according to the rule of Table 1. When the vehicle is reversing, the multi-sensor’s environmental perception module starts to detect and track rear obstacles to get the obstacle’s distance information in real time. In the simulation, a man suddenly appears 15 m behind the vehicle, but the driver keeps reversing the vehicle at 18 km/h without noticing him. In order to avoid a collision, the speed of the vehicle must be restricted according to the obstacle distance, as Figure 23 shows. 

As shown in Figure 23a,b, when the distance between the vehicle and the man is about 10 m, by obstacle detection and tracking, the vehicle begins to slow down from 18 km/h to 10 km/h. When the distance between the vehicle and the man decreases to 5 m, the vehicle automatically slows down to 6 km/h. When the driver continues to reverse the vehicle to 2.5 m away from the pedestrian, the vehicle speed drops to 2 km/h. Finally, when the distance is less than 0.4 m, the vehicle automatically begins to brake to zero to prevent a reversing accident. So with the assistant of the multi-sensors environmental perception using low-rank representation and particle filter, the driver can operate the automobile more safely and stably to prevent reversing accident.

## 7. Conclusions

In this paper, we present a novel multi-sensor environmental perception method using low-rank representation and a particle filter for vehicle reversing safety. The proposed system consists of four main steps, namely multi-sensor environmental perception, target recognition, target tracking, and vehicle reversing speed control modules. The final system simulation and practical testing results demonstrate the validity of the proposed multi-sensor environmental perception method using low-rank representation and a particle filter for vehicle reversing safety. This system has been tested on a DODGE SUV and a homemade vehicle. The theoretical analysis and practical experiments show that the proposed system not only has better performance in obstacle tracking and recognition, but also enhances the vehicle’s reversing control. The information fusion and particle filter tracking improve the accuracy of measurements and rear object tracking. The effectiveness of our system is one of the key factors for the active safety system; it can help reduce drivers’ work and tiredness and, accordingly, decrease traffic accidents. 

## Figures and Tables

**Table 1:** Various judgments of ECU under different conditions.

Distance	Acceleration Pedal	ECU Judgments
	Dramatic Accelerate	Emergency Braking
>10 m	Normal	Slow Down to 18 km/h
10 m–5 m	Normal	Warning & Slow Down to 10 km/h
5 m–2.5 m	Normal	Warning & Slow Down to 6 km/h
2.5 m–0.4 m	Normal	Warning & Slow Down to 2 km/h
<0.4 m	Normal	Braking to Zero

**Table 2:** Comparison of the algorithms.

Number of Frames	Algorithm Offered in [32] (fps)	Our Proposed Tracking Algorithm (fps)
30	20.0259	148.1246
60	18.0466	134.7846
90	18.3450	139.8128
120	19.5366	143.6594
150	16.7561	148.9651
180	15.3057	140.2364
210	15.3494	149.3074

**Table 3:** Tracking accuracy of the algorithms.

Video	Algorithm Offered in [32]	Our Proposed Tracking Algorithm
Car4	100%	100%
Car2	100%	100%
Walking	100%	100%
Video captured by ourselves (without shelter)	95%	96%
